# Beyond Immediate Responses: A Systems Map of Community Factors Underlying Persistent Gun Violence in New Haven

**DOI:** 10.1007/s11524-026-01123-w

**Published:** 2026-08-17

**Authors:** Sarah J. Ho, Zeynep Hasgul, Virginia T. Spell, Thomas A. Thornhill, Nadine Horton, Yiran E. Liu, Emily A. Wang, Mohammad S. Jalali, Brita Roy

**Affiliations:** 1 Yale School of Medicine, New Haven, CT USA; 2 Center for Health Technology Assessment, Mass General Brigham, Harvard Medical School, Boston, MA USA; 3 Urban League of Southern Connecticut, New Haven, CT USA; 4 Yale School of Public Health, New Haven, CT USA; 5 SEICHE Center for Health and Justice, Yale School of Medicine, New Haven, CT USA; 6 Yale Law School, The Justice Collaboratory, New Haven, CT USA; 7 Division of Health and Behavior, Department of Population Health, Section for Health Equity, New York University Grossman School of Medicine, New York, NY USA; 8 Division of General Internal Medicine and Clinical Innovation, Department of Medicine, New York University Grossman School of Medicine, New York, NY USA

**Keywords:** Gun violence, Community gun violence, Violence prevention, Gun violence prevention, Group model building

## Abstract

Gun violence is a pervasive and multifaceted public health problem. This study describes a novel application of group model building—a process that centers stakeholder voices to describe a complex problem—to investigate drivers of gun violence and design community-led interventions for prevention in New Haven, Connecticut. We recruited community leaders to participate in 2 group model building sessions in New Haven. Discussions and diagrams created during the sessions were used to produce a causal loop diagram illustrating proposed relationships between gun violence and other variables. Our resulting “iceberg model” illustrates how short-term and visible responses to gun violence “above the surface,” such as incarceration and policing, may only transiently decrease gun violence. Long-term, downstream effects of “above the surface” responses may ultimately increase gun violence and are summarized in 3 layers “under the surface,” which examine the effects of incarceration and lack of re-entry support, vulnerability to stress and social stigmatization, and community-police relations and community safety, respectively. This study demonstrates the value of using group model building to design community-driven interventions for gun violence prevention, as well as reiterates the importance of targeting structural causes of gun violence. This work has informed TRUE HAVEN, a multi-level intervention that addresses community gun violence in New Haven.

**Supplementary Information:**

The online version contains supplementary material available at https://doi.org/10.1007/s11524-026-01123-w.

## Introduction

Gun violence is a major public health problem, accounting for more than 48,000 deaths in the U.S. annually [1]. Consequences of gun violence are more likely to be experienced by socioeconomically disadvantaged and minoritized racial groups. Black Americans have a nearly 14-fold greater risk of firearm-related death compared to white Americans, and Black American males ages 15 to 34 years old comprise 36% of all gun homicide victims despite only comprising 2% of the general population [2]. Hispanic/Latino people and American Indian/Alaska Native people also experience higher rates of gun homicide than their white counterparts [2]. Beyond firearm-related mortality, the manifold impacts of gun violence affect survivors and their families and include increased rates of pain disorders, psychiatric disorders, and substance use disorders [3].

Gun violence is not solely an individual or interpersonal phenomenon, but rather, may be better understood within the community context, which encompasses the people who live in a certain geographic area as well as their interpersonal relationships, socioeconomic resources, historical legacy, and the community’s physical characteristics. Gun violence is concentrated in under-resourced communities that experience high levels of poverty and income inequality; it is also more prevalent in communities that have been subject to redlining [4, 5]. Commercial investment in a community may also influence gun violence, as increased numbers of vacant properties are associated with higher rates of violence, while areas with street lighting, crosswalks, public transportation, and parks are linked to lower rates of violence [4, 6].

Prior studies have investigated community-based interventions for reducing gun violence. The Cure Violence model is a public health approach that relies upon “violence interrupters” and outreach workers who establish one-on-one relationships with individuals who are at risk of perpetuating gun violence, helping to mediate conflict and introduce non-violent means of conflict resolution [7]. The Cure Violence model has been implemented in many cities, and while this intervention has reduced gun violence in some cities, it has not been uniformly successful, as the model has been difficult to implement and has overall achieved mixed results [7, 8]. The Comprehensive Gang Model, implemented widely by the federal government, centers collaboration between community members, social services agencies, and governmental agencies and has shown similarly mixed efficacy [9]. Another approach to mitigating rates of gun violence is focused deterrence led by law enforcement, which identifies the individuals and groups most involved in violence and uses a combination of deterrence strategies and coordination with community-based organizations to divert these individuals from further engaging in violence [10]. Focused deterrence has been implemented through initiatives such as Boston’s Operation Ceasefire, and has been broadly shown to be linked to meaningful reductions in gun violence [11, 12].

New Haven is a medium-sized city in Connecticut. Between 2011 and 2020, New Haven’s six predominantly Black and Latino neighborhoods experienced at least 70 shootings per 100,000 people—more than 15 times the national rate [13]. Forty-seven percent of adult New Haven residents reported being afraid of gun violence, and 13% witnessed a shooting in the past year [14]. New Haven is home to many community assets that are linked to lower rates of gun violence, including public green spaces, social services programs providing mental health and housing assistance, as well as programs focused on gun violence prevention. Programs aimed at reducing gun violence locally include Connecticut Violence Intervention and Prevention, which is based on the Cure Violence model [15]. In 2014, a $1 million government grant was awarded to New Haven from the U.S. Department of Justice to improve safety and decrease crime. New Haven has also been the site of interventions such as Project Longevity, a focused deterrence collaboration between the local police department, social service organizations, and community members, which has been associated with a reduction in gun violence [16]. Since 2011, rates of gun violence in New Haven have fallen, likely in part due to the combined efforts of the various gun violence interventions in the city [17]. However, rates of gun violence have plateaued and begun to rise in recent years—and New Haven still has the highest violent crime rate in Connecticut, suggesting additional and complementary strategies are needed for further improvement [18].

While focused deterrence interventions such as Project Longevity have been associated with reductions in gun violence, they work “downstream,” identifying individuals after an arrest associated with violence and aiming to prevent recurrence. Moreover, although community collaboration is integral to the success of such violence reduction programs, Project Longevity is led by law enforcement, and there have been few community-led gun violence interventions in New Haven [19]. Thus, in this study, we worked with the leadership of existing violence intervention programs, local community leaders, and those with lived experience of gun violence (victims and perpetrators) to identify opportunities to address “upstream” or root causes of gun violence for primary prevention. We engaged this group in a series of group model building (GMB) sessions to better understand New Haven’s unique context that perpetuates high rates of violence with the goal of designing feasible and context-appropriate interventions to prevent gun violence. GMB is a process that centers stakeholders and allows community leadership to better understand complex problems and identify opportunities for intervention [20]. A common product of GMB is a causal loop diagram (CLD), which depicts relevant variables in relation to each other and the problem of interest. Compared with other qualitative methods, GMB facilitates more structured and thorough discussions, reduces power gradients among participants, and improves communication and consensus [21]. By centering stakeholder perspectives, GMB is meant to improve the accuracy, acceptance, and ultimate implementation of the resulting model [22]. GMB has previously been applied to investigate structural racism, youth violence, and racial experiences of community violence [23, 25]. In our study, we conducted a series of GMB sessions with community stakeholders in New Haven to better understand the multilevel processes underlying community gun violence in New Haven and elucidate potential interventions.

## Methods

This study was reviewed and approved by the Yale University Institutional Review Board (protocol number 2000022360).

### Recruitment

This study is part of a broader collaboration between community members and academic researchers that employs a multi-tiered approach to understanding and designing interventions for gun violence in New Haven [26]. As of 2025, New Haven is a city with a population of 135,000, including 36.7% white residents, 30.6% Black residents, and 31.2% Hispanic or Latino residents. The median household income is $53,700, and 25% of residents live in poverty [27]. Prior to this study, this group of community members and academic researchers had adapted and applied RAND’s framework for disaster preparedness, “Building Community Resilience to Disasters,” using a multi-pronged approach to build community resilience in response to gun violence [26, 28]. Purposive sampling and existing connections with community partners were leveraged to invite additional local leaders representing each of the 8 levers of the RAND Community Resilience Framework to participate in the GMB sessions [28]. All participants, with the exception of Yale University personnel, were compensated for their participation via mailed checks for $250 per person.

### GMB sessions

GMB involves a trained facilitation team that works with stakeholders to iteratively develop and refine a CLD that visualizes the relationships among multiple factors associated with the complex problem over time [22]. GMB facilitators use scripts to guide and direct discussion, such that all participants can contribute [22, 29].

Two GMB sessions took place and were led by a main facilitator who had completed training in GMB facilitation, complex systems science, and systemic dynamics modeling. The sessions were supported by a convener/closer, a community facilitator/gatekeeper, a modeler/model facilitator, a process coach/timekeeper, wall-builders and assistant data managers, and a recorder/data manager/report generator.

Each GMB session was in person and lasted approximately 4 hours. Standard GMB scripts were used to facilitate the sessions [29]. The first session followed a set agenda to elicit variables and begin to develop a CLD (Supplemental Table S1). After introductions, an overview of the project’s objectives, and an icebreaker, scripts were used to establish a safe space for collaboration (a “Gracious Space”) and to introduce GMB notation and diagramming convention [25]. Participants were then asked to independently draw a graph over time of their perception of the number of people affected by gun violence in New Haven, what they expected would happen in the future if no intervention occurred, and what they hoped to see instead [30]. Using these graphs, a consensus graph, or a reference mode, was reached [31]. Next, using a Round Robin technique to facilitate equal participation, participants generated a list of variables that influence community gun violence. Throughout the rest of the first session and in the second session, variables were iteratively incorporated into a single CLD with directional arrows signifying the hypothesized relationship between factors, which was revised until consensus was reached [32].

### CLD analysis

Following the development of the CLD during the GMB sessions, we reviewed the diagram to identify key feedback loops and their interactions. The CLD includes reinforcing and balancing feedback loops (denoted in subsequent figures as R and B, respectively). Reinforcing loops represent feedback structures that amplify change in the system over time. Balancing loops represent stabilizing structures that work to counteract change and restore equilibrium within the system.

Our analytic goal was to assess how different factors and loops might contribute to the persistence or reduction of community gun violence. Insights were drawn by tracing how variables connected across loops and by reflecting on recurring structures that could inform intervention design. These reflections exposed system-level dynamics and opportunities for action that were not immediately apparent from individual variables alone.

## Results

Nineteen local stakeholders were recruited, including those representing the housing, education, law enforcement, academic, community leadership, youth program, mental health, crisis outreach, and incarceration sectors. Specifically, GMB participants included leadership from Project Longevity, local youth mentoring programs, and mental health clinics. Participants included those who identified as Black, Hispanic, and White, and included both men and women. Participant ages ranged approximately from 30 years old to 60 years old. Among the participants were those with a history of incarceration, those impacted by gun violence, as well as those who owned guns and did not own guns. All 19 participants attended both GMB sessions. Preliminary CLDs showing proposed relationships between variables were created during GMB sessions (Supplemental Figure S1). The resultant CLD was subsequently refined and simplified for reporting purposes and ease of understanding; simplification involved consolidating conceptually similar variables and removing peripheral variables that did not materially alter the core feedback structure identified during the workshops. This refined version was endorsed by a subset of the original GMB group. The final CLD reflects a system of drivers and downstream consequences of community gun violence that operates across multiple layers. We present this model using the analogy of an iceberg, wherein the upper layer, i.e., the “tip of the iceberg,” represents reactive responses that are more immediate and visible. “Below the surface” layers represent CLD components that are less visible and often unfold in the long term.

### Above the Surface: Punitive Control

**Fig. 1:**
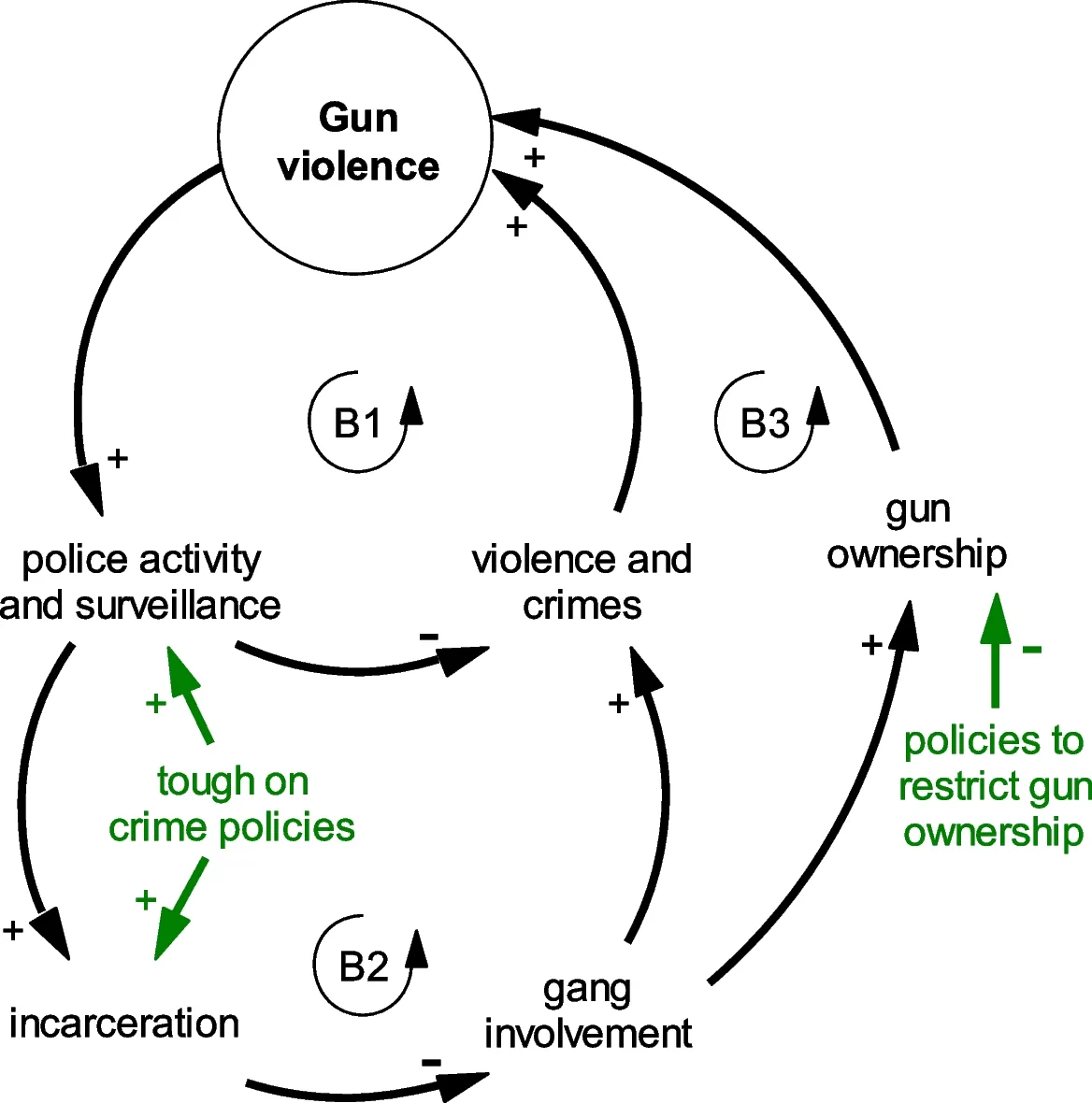
Above the Surface: Above-the-surface structure of the CLD, representing reactive responses to gun violence through loops B1–B3. Arrows between CLD components represent relationships identified by stakeholders. “B” indicates balancing loops. Green variables denote policy levers. Gun violence is shown in a circle to represent our main focus. Figure created with Vensim DSS, version 10

The upper layer of the model includes policing strategies, carceral responses, and regulatory interventions aimed at the containment of gun violence, illustrated through 3 balancing feedback loops (Fig. 1). In this model, gang involvement is proposed to increase violence and crimes as well as gun ownership, both of which drive gun violence. Participants described gun violence as leading to increased police activity and surveillance, which is intended to reduce violence and crime and subsequently decrease gun violence (B1). Greater police activity was also proposed to result in more incarceration, reducing gang involvement and gun violence (B2). Tough-on-crime policy initiatives reinforce these 2 feedback loops. Within the group, which included both gun owners and non-gun owners, participants identified gun ownership, influenced both by guns entering their community from outside suburban areas as well as the strictness of gun laws, as being an important driver of gun violence. As gun violence increases, policy responses aimed at restricting gun ownership seek to reduce access to firearms, thereby lowering the likelihood of future violence (B3).

This above-the-surface portion of the model aims to stabilize the system by reducing gun violence directly. While these loops may temporarily reduce violence, they risk reinforcing structural dynamics that sustain violence over time. To support readability and interpretation, we next present the below-the-surface components of the CLD in 3 separate layers, each focusing on a subset of reinforcing loops. In each successive figure, previously discussed portions of the diagram are grayed out to help focus on the newly introduced structures.

### Below the Surface I: Reentry, Social Needs, and Gang Dynamics

**Fig. 2:**
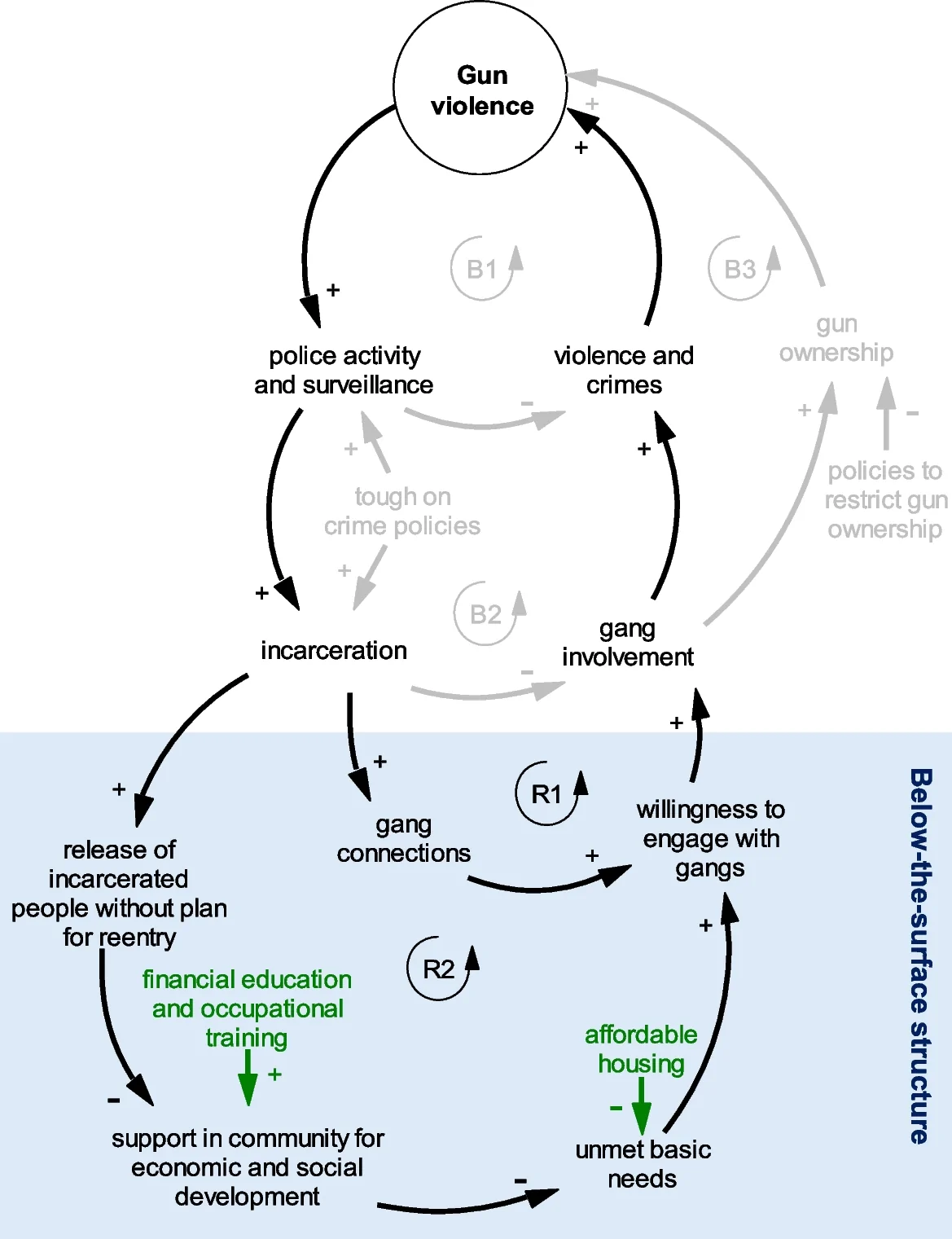
Below the Surface I: Below-the-surface structure of the CLD, presenting reinforcing loops (R1–R2) related to gang connections and unmet needs. “R” represents reinforcing loops. Figure created with Vensim DSS, version 10

While incarceration may decrease gun violence in the short term, in the long term, it may actually increase gun violence through less immediate and less visible dynamics (Fig. 2). Participants noted that gang connections play a role both inside and outside the carceral system, especially because individuals incarcerated for non-shooting offenses may form gang connections in prison. Therefore, while incarceration may initially decrease gang involvement (B2), it ultimately may facilitate more gang connections through relationships formed while incarcerated (R1). These strengthened connections may increase willingness to engage with gangs after release, which can further contribute to violence and future incarceration, reinforcing the cycle over time.

Participants also cited gang membership as representing a survival strategy and a means of building community and safety. Often, individuals are released from incarceration without a reentry plan or support; subsequently, many face unmet basic needs, such as unstable housing, economic insecurity, and limited access to mental health care. As described by participants, these unmet needs and lack of support increase individuals’ vulnerability and willingness to join gangs (R2). Strengthening social support systems for people released from incarceration, including financial education, job training, and access to affordable housing, may help reduce this vulnerability and interrupt reinforcing dynamics.

### Below the Surface II: Stress, Stigma, and Escalation

**Fig. 3:**
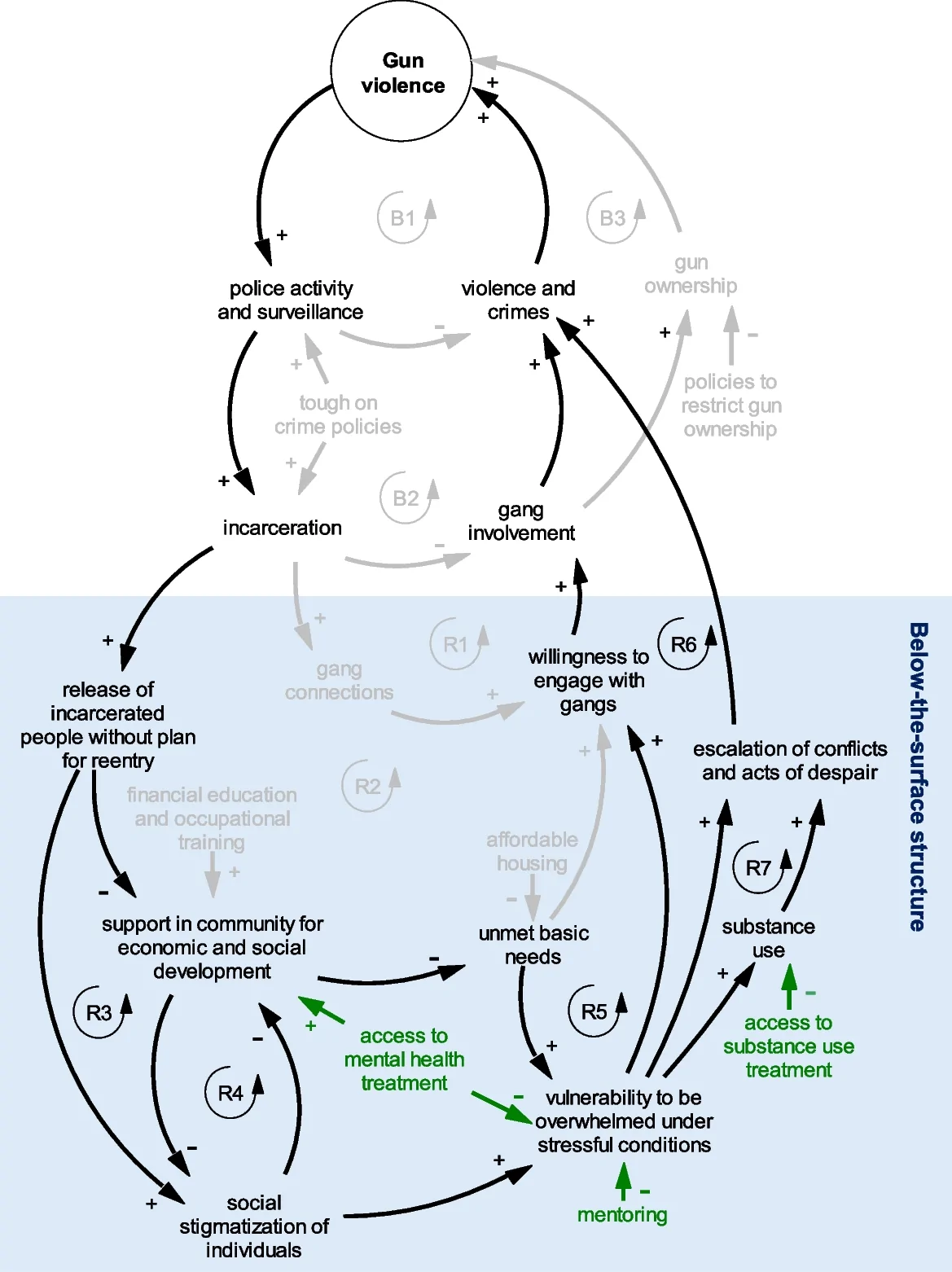
Below the Surface II: Expanded structure of the CLD, illustrating deeper reinforcing loops (R3–R7) related to stigma, stress, and escalation of conflicts. Figure created with Vensim DSS, version 10

Figure 3 extends the model further below the surface, capturing how structural disadvantages, stigma, and stress responses reinforce cycles of gun violence. Without reentry support, individuals who are released from incarceration often encounter social stigmatization (R3), as there is a vast system of laws that prevent people with criminal records from obtaining publicly funded services and re-entering the labor force. This stigmatization can limit an individual’s economic and social development, which in turn reinforces stigma (R4) and can render individuals more vulnerable to external stressors. This vulnerability can be further exacerbated by the unmet basic needs that individuals without the necessary reentry support face (R5). This increased vulnerability may then lead to escalated conflict, acts of despair, and involvement in gun violence, feeding back into the cycle of gun violence and incarceration (R6). Many participants cited mentoring as a way to support educational achievement, define positive norms, and serve as a protective factor against gang involvement in situations of increased vulnerability. Participants also reflected on the importance of mental health and that access to mental health care is complicated by stigma, financial barriers, and a lack of providers who can empathize with what participants have experienced. A combination of this lack of access to mental health resources and increased vulnerability to stress can also lead to substance use; participants pointed to substance use, and alcohol use in particular, as increasing gun violence and physical injury (R7). Accordingly, participants identified access to mental health care, substance use treatment, and mentorship as ways to interrupt these feedback loops and reduce the likelihood of escalation.

### Below the Surface III: Safety, Trust, and Community Cohesion

**Fig. 4:**
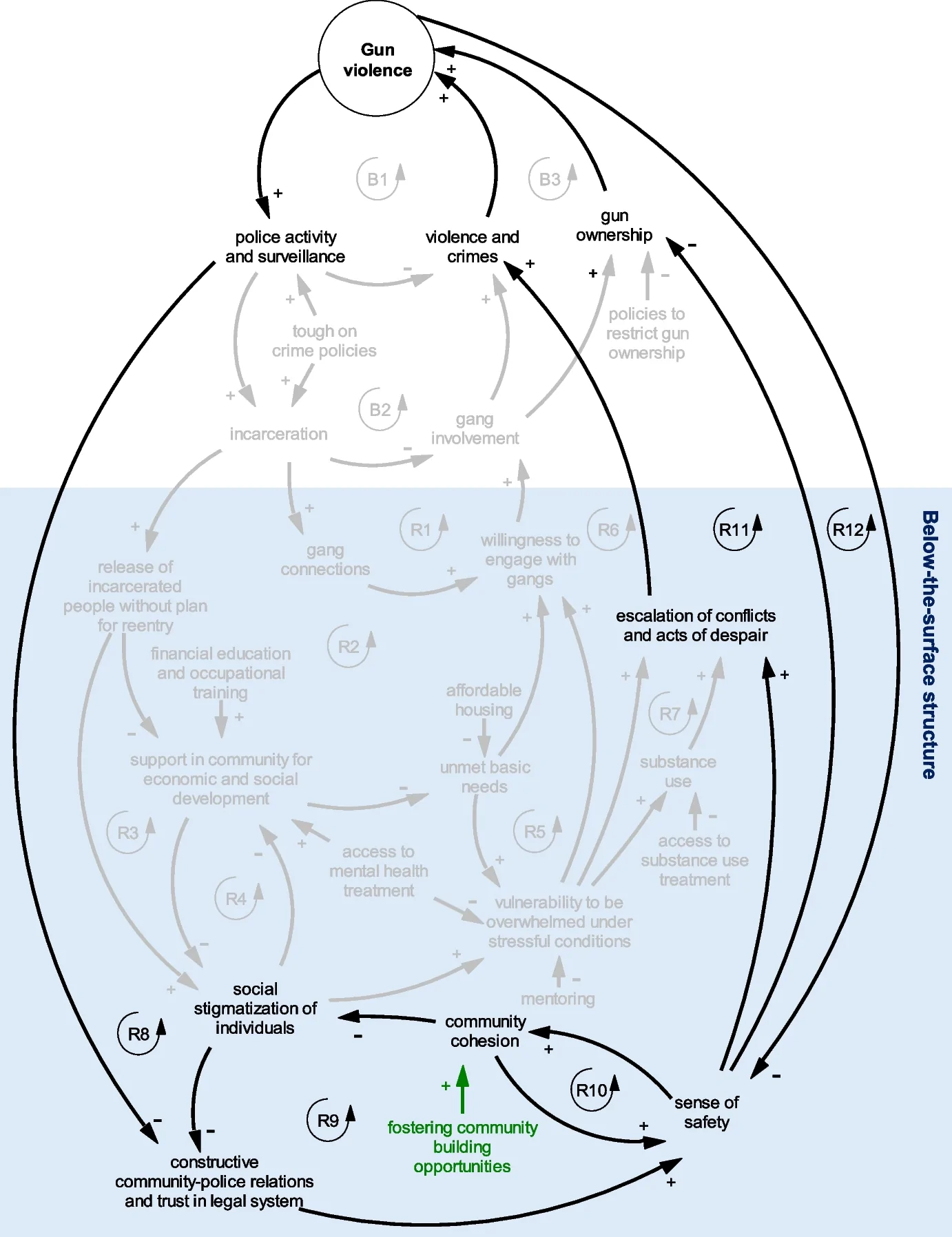
Below the Surface III: Expanded CLD showing deeper reinforcing loops (R8–R12) related to safety, community cohesion, and firearm dynamics. Figure created with Vensim DSS, version 10

Figure 4 presents an additional layer of below-the-surface dynamics, focusing on how community cohesion, trust, and perceptions of safety are shaped by and shape patterns of gun violence. Increased police activity and the stigmatization of individuals with a history of incarceration were proposed by participants to erode constructive relationships between the community and law enforcement, weakening the community’s trust in the legal system (R8, R9). Worsened community-police relations, in turn, lower the sense of safety and community cohesion (R9), with participants noting that a lack of trust in the legal system often led to people resorting to extralegal ways to settle disputes. Moreover, participants discussed how a reduced sense of safety itself can weaken community cohesion, which further diminishes feelings of safety (R10), as residents did not feel safe enough to take walks in their neighborhood, allow their children to play outside, or talk to their neighbors.

Participants described lower levels of perceived safety as increasing individual gun ownership and subsequently gun violence (R11). This, in turn, could increase police surveillance and incarceration rates, further weakening community-police relations and trust in the legal system, reducing the sense of safety even further. Loop R12 captures a more direct reinforcing pathway as proposed by participants: increases in rates of gun violence reduce the sense of safety, prompting greater gun ownership, which then fuels more gun violence. Together, these loops emphasize how fear and perceived insecurity can activate feedback structures that sustain and escalate violence in communities. Importantly, these effects do not just impact individuals involved with gun violence but radiate outward to impact the community as a whole.

## Discussion

Our GMB sessions revealed that more immediate and reactive responses to curtail gun violence may actually have the opposite effect, creating conditions that perpetuate community gun violence over the ensuing years and decades. Individuals released from incarceration with little-to-no reentry support contend with social stigmatization while struggling to gain socioeconomic stability. As a result of housing insecurity, financial instability, and lack of access to mental health resources, they are more likely to engage with gang membership and are more vulnerable to external stressors and substance use, all of which can contribute to more gun violence. Our model also demonstrates that policing and reactive responses to gun violence negatively impact the well-being of the community as a whole, worsening community-police relations, feelings of community cohesion, and perceptions of safety. In turn, decreased trust in the legal system and perception of lack of safety can lead to more gun ownership and gun violence. The model also contains several reinforcing loops, which suggests that these effects are likely amplifying and self-perpetuating across multiple levels—affirming the observation that in New Haven, despite an abundance of programs addressing gun violence (e.g., violence intervention and prevention programs; heightened policing; gun buyback programs), rates of gun violence still remain high. Importantly, in our CLD, all of the loops in the “Above the Surface” layer are balancing loops, demonstrating how short-term and overt responses to gun violence seek to immediately address precipitating factors, while all of the loops in the “Below the Surface” layers are reinforcing loops, exemplifying that these relationships and mechanisms, when developed over longer time scales, can insidiously act to amplify the problem of gun violence.

We find that when structural supports, trust, and safety are strengthened, the same reinforcing loops can operate in opposite directions and can become virtuous cycles, amplifying positive change and reinforcing conditions that reduce violence over time. Although we presented the model beginning with gun violence, it can just as easily be read from the bottom up, showing how a lack of essential supports, such as housing and mental health care, creates conditions where violence becomes more likely. In other words, the system itself produces the very risks it later seeks to control. Strengthening those foundational supports offers a pathway to interrupt these patterns and reduce violence at its roots. These findings are further validated by results from a qualitative study also conducted in New Haven, in which thematic analysis of semi-structured interviews among a separate, larger cohort of local community members found that home ownership, social cohesion, community support, and access to mental health services were perceived as important factors for preventing gun violence [33]. Our CLD elucidates how such factors influence the pathways leading to persistent violence in New Haven.

Discussions throughout the GMB sessions emphasized bolstering housing support and the strong presence of mental health providers within New Haven as new opportunities for community-led interventions to prevent gun violence. Similarly, schools and prisons were also seen in the model as potential areas of intervention, but the lack of engagement from the public education sector and the difficulty of implementing a community-led intervention within carceral facilities, respectively, made these sectors difficult to incorporate into the intervention design. Our findings from the GMB sessions played a central role in informing the components of a multi-level intervention to reduce gun violence in New Haven: Trusted Residents and Housing Assistance to Decrease Violence Exposure in New Haven (TRUE HAVEN). From the GMB sessions, five potential interventions with high feasibility and likelihood of success were identified. Of those, two were selected based on the strength of existing partnerships and funding opportunities, leading to the development of the TRUE HAVEN intervention. TRUE HAVEN provides financial assistance, education, and housing support to people affected by incarceration on the individual level and trains community leaders on trauma-informed care on the neighborhood level [13]. Of note, this study is the first to date to use GMB to design an intervention to mitigate the rates and effects of community gun violence. Further, the GMB sessions themselves helped deepen community partnerships, fostering community stakeholders’ readiness and ability to collaboratively design and implement TRUE HAVEN.

Our findings add to the emerging literature applying GMB to elicit the complex linkages among causes and consequences of community violence and structural racism over time [23–25]. GMB sessions held in a different community examining the issue of community violence similarly highlighted the contributions of gun ownership, social support, mental health, and housing security [25]. Our findings also align with literature that has established the far-reaching and socioeconomically constraining effects of incarceration, the importance of examining the legitimacy of the criminal legal system, the relationship between distrust in law enforcement and increased individual protective gun ownership, and the association of food, housing, and financial insecurity with violent crime [34–37]. Taken together, using GMB to explore the interconnected factors associated with community gun violence, we begin to see convergence among associated factors and their relationships across different small, urban settings. Further investigation identifying commonalities across communities and contextual factors that may yield different results is warranted. While many of the identified factors have been documented previously, the contribution of this works lies in the systems perspective that emerged from bringing together community knowledge to visualize the multilevel interactions and pathways that shape gun violence, thereby informing the development of a community-led intervention within a specific local context.

Many current interventions, both those implemented locally in New Haven and those implemented broadly, concentrate resources on individuals involved in gang membership while also striving to address structural and social factors. Our findings emphasize the continued need for interventions that address the root causes of community gun violence, such as financial education and occupational training after release from incarceration, housing security, mental health and substance use treatment, and mentorship to improve social cohesion. Investing in supporting individuals post-incarceration and the stability of communities may help comprehensively prevent gun violence in the long term. Moreover, by enhancing virtuous cycles (e.g., strengthening structural supports, trust, and safety) and disrupting negative reinforcing loops, this approach may have long-lasting positive impacts that amplify over time.

### Limitations

The GMB sessions could only accommodate a limited number of stakeholders, so our CLD may not represent all perspectives. The relationships depicted in the CLD represent stakeholder perceptions and should not be interpreted as empirically validated causal effects. All individuals recruited were aged 18 years and above, so we could not directly elicit the perspectives of children and adolescents, who are often most impacted by community gun violence. In addition, the social dynamics of an in-person group workshop may have precluded full participation from all members. To address this, we established participation guidelines during the first session and used established GMB scripts and exercises to promote participation among all group members. Lastly, because our study is focused on understanding community gun violence in New Haven, the generalizability of our findings to other communities and cities is unclear. However, related work in other communities aligns with our results, suggesting our findings are at least generalizable to other urban and small urban areas.

## Supplementary Information

Below is the link to the electronic supplementary material.

ESM 1 (535 KB DOCX)
