# Supplementary material for: Beyond Immediate Responses: A Systems Map of Community Factors Underlying Persistent Gun Violence in New Haven
Source: J Urban Health. 2026 Aug 17;103(5):1034–44. doi: 10.1007/s11524-026-01123-w (PMC13672730; doi:10.1007/s11524-026-01123-w)
Supplement: Supplementary file 1 [file 11524_2026_1123_MOESM1_ESM.docx]

Supplement

Table S1: Agenda for the first GMB session.

| **Time** | **Activity** | **Description** |
| --- | --- | --- |
| 15 minutes | -Lunch  -Initial set-up | Everyone gets lunch. Name tags, materials, and W-9s distributed. |
| 10 minutes | -Welcome  -Introductions | Provide your name, your organization, and your current mood in one word |
| 20 minutes | -Icebreaker | Count off by 3’s and get into groups. List as many things as you can think of that you have in common. |
| 15 minutes | -Session introduction  -Gracious Space | Introduction to the purpose of the GMB sessions. Discussion of the Gracious Space, with guidelines written down on a large piece of paper |
| 15 minutes | -Presenting the reference mode  -Defining the reference mode  -Script used: [Presenting Reference Mode](https://en.wikibooks.org/wiki/Scriptapedia/Presenting_the_Reference_Mode) | Use an example to demonstrate how to draw a reference mode. Ask each participant to draw a graph for the number of people affected by gun violence, what they expect will happen over time, and what they would like to see instead. Participants will then post their drawings on the wall. Similarities and differences between graphs will be discussed. After the group comes to a consensus, the consensus reference mode will be drawn and posted. |
| 15 minutes | -Orientation to Casual Loop Diagram notation | Arrow and loop notation will be posted on the wall. |
| 20 minutes | -Variable elicitation | Participants are asked to individually list as many conditions that contribute to or mitigate rates of community gun violence as possible. These can be positive or negative, assets, resources, or outcomes related to gun violence. Round-robin elicitation of variables for two rounds, keeping track of variables on an easel pad. |
| 10 minutes | -Categorize variables | Organize variables into categorized lists, and copy these new categorized lists onto the easel pad. |
| 15 minutes | Break |  |
| 90 minutes | -Building causal loop diagrams  -Script used: [Initiating and Elaborating CLDs](https://en.wikibooks.org/wiki/Scriptapedia/Initiating_and_Elaborating_a_Causal_Loop_Diagram) | Everyone is given 3 votes (3 stickers) and asked to put them next to the variable they think is the most important. Will then start to add elicited variables in connection with the main issue (community gun violence). |
| 30 minutes | -Recap  -Consensus  -Reflection  -Next steps  -Closing remarks | Step back from the causal loop diagram and evaluate for consensus. After reflection, review the next steps. |

Figure S1a-c: Sequential preliminary CLDs created during GMB sessions.

(a)

(b)

(c)
